# Efficacy and safety of PD-1/PD-L1 and CTLA-4 immune checkpoint inhibitors in the treatment of advanced colorectal cancer: a systematic review and meta-analysis

**DOI:** 10.3389/fimmu.2024.1485303

**Published:** 2024-11-01

**Authors:** Dandan Song, Shufu Hou, Ning Ma, Bing Yan, Jing Gao

**Affiliations:** ^1^ Department of Neurology, Shandong Provincial Third Hospital, Cheeloo College of Medicine, Shandong University, Jinan, China; ^2^ Shandong Provincial Third Hospital, Cheeloo College of Medicine, Shandong University, Jinan, China; ^3^ Department of Gastrointestinal Surgery, Central Hospital Affiliated to Shandong First Medical University, Jinan, China; ^4^ Department of General Surgery, The First Affiliated Hospital of Shandong First Medical University, Jinan, China

**Keywords:** immune checkpoint inhibitors, colorectal cancer, immunotherapy, PD-1, PD-L1, CTLA-4

## Abstract

**Background:**

The efficacy and safety of PD-1/PD-L1 inhibitors combined with CTLA-4 inhibitors in the treatment of advanced colorectal cancer is controversial. This meta-analysis aimed to evaluate the efficacy and safety of PD-1/PD-L1 inhibitors combined with CTLA-4 inhibitors for advanced colorectal cancer.

**Methods:**

PubMed, Embase, the Cochrane Library, and Web of Science databases were systematically searched for relevant studies. Outcomes including median progression-free survival (mPFS), median overall survival (mOS), overall response rate (ORR), disease control rate (DCR), treatment-related adverse events (TRAEs) and ≥grade 3 TRAEs were extracted for further analysis. The risk of bias was assessed by subgroup analysis.

**Results:**

12 articles with 566 patients were identified and subjected to meta-analysis. With regard to survival analysis, the pooled mOS and mPFS were 6.66 months (95%CI 4.85-9.16) and 2.92 months (95%CI 2.23-3.83), respectively. In terms of tumor response, the pooled ORR and DCR were 21% (95%CI 6%-41%) and 49% (95%CI 27%-71%), respectively. The pooled AEs rate and ≥ grade 3 AEs rate were 94% (95%CI 86%-99%) and 44% (95%CI 30%-58%).

**Conclusion:**

PD-1/PD-L1 inhibitors combined with CTLA-4 inhibitors have shown promising clinical responses in the treatment of colorectal cancer (CRC). Although the incidence of adverse reactions is high, they are generally tolerable.

**Systematic review registration:**

https://inplasy.com/, identifier INPLASY202480030.

## 1 Introduction

Colorectal cancer (CRC) is the third most commonly diagnosed malignancy worldwide and the fourth leading cause of cancer-related death (1, 2). Its global incidence is expected to rise to 2.5 million new cases by 2035 (3, 4). CRC development results from a complex interplay of genetic and environmental factors, including a sedentary lifestyle, age, obesity, and dietary habits (4–8). Due to its insidious onset, approximately 20% of cases are diagnosed as metastatic colorectal cancer (mCRC) (9), and about 40% of patients with localized disease experience recurrence after prior treatment (10).The high mortality rate in advanced colorectal cancer (aCRC) is largely due to tumor progression and metastasis. Despite advances in treatment, the prognosis for aCRC patients remains poor, with a median overall survival (mOS) of 30 months (11, 12) and a 5-year survival rate of less than 14% (13, 14). Surgery, chemotherapy, and radiotherapy remain the primary therapeutic interventions for CRC, often used in combination depending on disease localization and progression (15–18). Surgery is the cornerstone of curative treatment for localized CRC (stage I–III) (19), and in mCRC with limited metastasis, surgery and chemotherapy may achieve curative outcomes in some cases (14, 19, 20). However, complete cancer cell removal is often unattainable, leading to disease recurrence in many patients (21). Although significant progress has been made in systemic and local therapies for aCRC, it remains rarely curable. Resistance to conventional treatments, systemic toxicity, and low selectivity highlight the need for more effective alternatives (13, 21, 22).

Immunotherapy has emerged as a promising approach, aiming to reverse tumor-induced immune suppression and activate antitumor responses (23). Immune checkpoints, such as programmed cell death 1 (PD-1), its ligand (PD-L1), and cytotoxic T lymphocyte antigen 4 (CTLA-4), have been extensively studied because of their overexpression in many solid tumors and hematological malignancies (24, 25). Blocking the interaction between PD-1/PD-L1 or CTLA-4 and their ligands activates T cells, promoting tumor infiltration and cancer cell death (26, 27). In response, the U.S. Food and Drug Administration (FDA) approved immune checkpoint inhibitors (ICIs) for patients with microsatellite instability-high (MSI-H) or mismatch repair-deficient (dMMR) CRC. These cancers exhibit high mutation rates and a strong neoantigen burden, triggering robust T cell-mediated tumor immune responses (28–30). A recent study showed that 100% of 12 patients with advanced dMMR rectal cancer achieved a complete clinical and pathological response after six months of treatment with dostarlimab (31). However, ICIs have limited efficacy in proficient mismatch repair (pMMR), microsatellite-stable (MSS), or microsatellite instability-low (MSI-L) CRC, which account for 85% of cases (32, 33). Given the biological complexity of the tumor microenvironment (TME) in most solid tumors, a multi-checkpoint inhibition strategy seems logical (34). Since CTLA-4 and PD-1 target non-redundant pathways, combining inhibitors for both may provide additive or synergistic effects. Some clinical trials have reported improved outcomes with combined PD-1/PD-L1 and CTLA-4 blockade compared to monotherapy (35). However, other studies suggest that combining these inhibitors has shown limited success in treating CRC (36). Moreover, combined ICI therapy is associated with a higher incidence of adverse events compared to single-agent treatment (37). To address these uncertainties, we conducted a systematic meta-analysis of the existing literature to evaluate the efficacy and safety of combining PD-1/PD-L1 and CTLA-4 inhibitors in CRC treatment.

## 2 Methods

### 2.1 Article searching

This meta-analysis was conducted in strict accordance with the guidelines outlined in the Preferred Reporting Items for Systematic Reviews and Meta-Analyses (PRISMA) checklist (38), which ensures comprehensive, transparent, and methodologically sound reporting of systematic reviews and meta-analyses. The study protocol has been registered with the International Platform of Registered Systematic Review and Meta-analysis Protocols (INPLASY) under Registration ID: INPLASY202480030.We performed a thorough search of online databases, including the Cochrane Library, Embase, and PubMed, for relevant clinical trials published up until April 13, 2024. The search terms included “anti-PD-1,” “anti-PD-L1,” “anti-CTLA-4,” “immune checkpoint inhibitors,” “colorectal cancer,” “CRC,” “mCRC,” and “aCRC.” Both free-text terms and Medical Subject Headings (MeSH) were used to search within titles and abstracts. Additionally, the reference lists of selected articles were screened to ensure comprehensive coverage. In cases of duplicate publications, the most comprehensive studies were selected for inclusion in the meta-analysis. Two authors independently extracted all relevant data, and any discrepancies were resolved through discussion and consensus.

### 2.2 Study selection

Obtained records were exported to EndNote software (Clarivate Analytics, Philadelphia, PA, USA).After removing the duplicate publications, two review authors independently reviewed the title/abstract of the articles according to the inclusion and exclusion criteria. Afterward, the same two authors screened the full-texts of the selected records, independently. Discrepancies were resolved by consulting a third author.

### 2.3 Eligibility criteria

Trials were included if the following criteria were met (1):patients with aCRC aged 18 years or older were enrolled; (2):a PD-1/PD-L1 and CTLA-4 inhibitors with or without other standard treatments was given to one of the study arms; and (3):outcomes of interest in terms of efficacy (i.e. median overall survival [mOS], median progression-free survival [mPFS], objective response rate [ORR], disease control rate [DCR]),and safety (i.e.AEs and ≥ grade 3 AEs)were reported.

The exclusion criteria were as follows: (1) Non-advanced CRC included early, middle and locally advanced CRC; (2) animal experiments, cell research, reviews, meta-analyses,duplicates, case reports, or letters were not taken into consideration; and (3) studies with patient number less than 10 were excluded. Two investigators independently identified potential eligible articles through inclusion and exclusion criteria. Any disagreement regarding study inclusion was resolved between these two or with a third investigator.

### 2.4 Data extraction and quality assessment

Two researchers conducted independent literature searches, following predetermined criteria and specified strategies. This approach ensures a thorough and unbiased exploration of available literature, utilizing a systematic and structured methodology. Meticulous data extraction was performed, encompassing essential details such as authors, publication year, country, trial duration, Identifier, sample size, gender, age, median follow-up, name of ICIs, Other therapy besides ICIs,molecular phenotype and survival analyses, including mOS, mPFS, DCR, ORR, AEs、≥ grade 3 AEs. All included studies were treated as non-randomized trials. The quality of each study was meticulously evaluated using the methodological index for non-randomized studies (MINORS) (39). Studies scoring above 12 points were considered high-quality indicators. This stringent evaluation ensures that only studies meeting robust methodological standards contribute to the overall analysis.

### 2.5 Data synthesis

The primary efficacy endpoint was to estimate the mOS and mPFS after receiving PD-1/PD-L1 inhibitors combined with CTLA-4 inhibitors treatment regimens and the secondary efficacy endpoint was to estimate the pooled rate of ORR and DCR. The safety outcome was the pooled rate of AEs and ≥ grade 3 AEs, We used Cochrane’s Q statistic to assess between-study heterogeneity and calculated the Isquare statistic. A random-effect model was applied if obvious heterogeneity was present (I2 >50%), otherwise, a fixed-effect model was chosen (40). The subgroup analysis was conducted according to country(USA, Other), sample(≥40, <40),other therapy(None,Yes), ICIs types (Nivolumab+Ipilimumab, Durvalumab+tremelimumab, QL1706 (PSB205)) and molecular phenotype (dMMR/MSI-H, pMMR/MSS, NR). Differences between groups were tested by the chi-square test. We used STATA version 18.0 (41) to calculate the pooled rates with metaprop command, which requires a nominator and a denominator (which is the total sample size) and some other options like random or fixed effects model. This command was built on the existing Stata command metan, which is routinely used to pool ratios and differences of means (42). A p-value less than 0.05 were treated as statistically significant.

## 3 Results

### 3.1 Study selection and characteristics of the included studies

**Figure 1:**
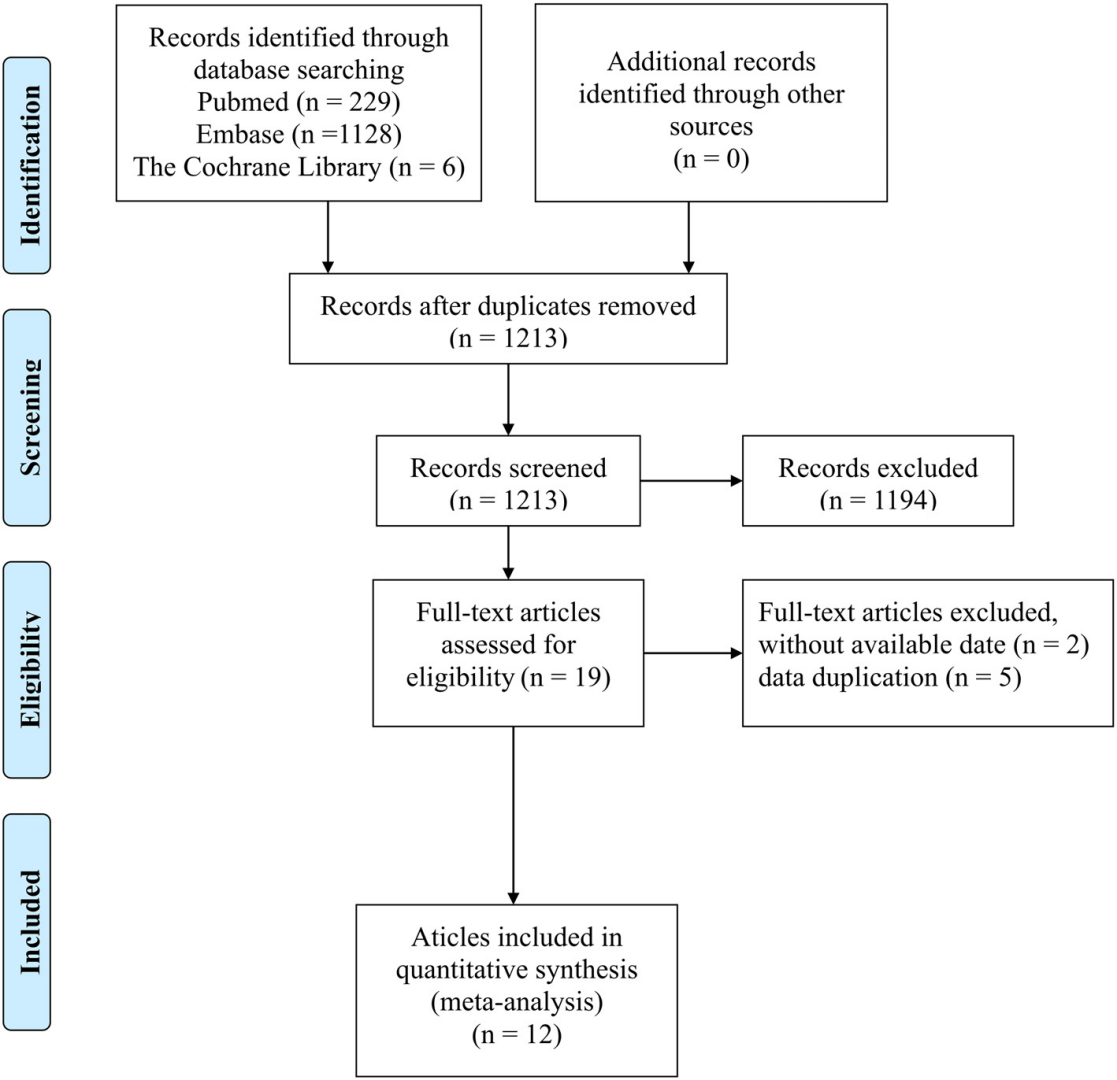
Prisma flowchart illustrating the literature selection process.

There were 1363 documents searched from the databases. Of these, 150 replicated studies were deleted. After reading the title and abstract of each article, 19 articles were screened out. The full texts of these articles were then assessed comprehensively. 12 articles (35, 36, 43–52)that meet the criteria were selected with a total of 566 patients. **Figure 1** summarizes the detailed information about article selection. The 12 and 11 included studies were eligible for ORR and DCR, respectively. The 8 included studies were eligible for mPFS and adverse reaction data analysis, and of those, 6 were eligible for mOS data analysis. **Table 1** lists the characteristics of the 12 trials. The extracted characteristics were summarized as follows: authors, publication year, country, trial duration, Identifier, sample size, gender, age, median follow-up, name of ICIs, Other therapy besides ICIs and molecular phenotype.

**Table 1:** Baseline characteristics of included studies.

Study, year	Country	Duration	Identifier	Sample size	Gender (M/F)	Age (range)	Median follow-up (months)	Name of ICIs	Other therapy besides ICIs	Molecular phenotype	MINORS score
André et al., 2022	USA	NR	NCT02060188	119	70/49	58 (21-88)	50.9	Nivolumab+Ipilimumab	None	dMMR/MSI-H	14
Chen et al., 2020	Canada	2016-2017	NCT02870920	119	74/45	65 (39-87)	15.2	Durvalumab+tremelimumab	None	pMMR/MSS	15
Cohen et al., 2020	France	2017-2018	NCT03350126	57	30/27	56.5(45.8-63.8)	18.1	Nivolumab+Ipilimumab	None	MSI/dMMR	15
Fakih et al., 2023	USA	2020-2022	NCT04362839	29	12/17	55 (36-75)	NR	Nivolumab+Ipilimumab	Regorafenib	MSS	14
Marie et al., 2021	USA	2016-2020	NCT02754856	23	12/11	56 (28–69)	27.6	Durvalumab+tremelimumab	None	pMMR/dMMR	15
Monge et al., 2023	USA	NR	NR	18	8/10	56.5 (28-76)	14	Durvalumab+tremelimumab	PexaVec	pMMR/MSS	13
Monjazeb et al., 2021	USA	NR	NCT02888743	20	9/11	59 (29-79)	3.9	Durvalumab+tremelimumab	Radiotherapy	MSS	12
Morano et al., 2022	Italy	2019-2020	NCT03832621	33	17/16	58 (53-65)	23.1	Nivolumab+Ipilimumab	Temozolomide	pMMR/MSS	14
Parikh et al., 2021	USA	NR	NCT03104439	40	22/18	59 (26-83)	16.6	Nivolumab+Ipilimumab	Radiotherapy	MSS	14
Segal et al., 2021	USA	NR	NCT03122509	24	13/11	55 (26-78)	21.8	Durvalumab+tremelimumab	Radiotherapy	pMMR	13
Thibaudin et al., 2023	France	2017-2019	NCT03202758	57	24/33	63.6 (28-80)	36	Durvalumab+tremelimumab	mFOLFOX6	MSS/MSI	15
Zhao et al., 2023	China	2020-2021	NCT04296994 and NCT05171790	27	NR	NR	9.5	QL1706 (PSB205)	None	NR	14

M, male; F, female; NR, not report; Nivolumab; Ipilimumab; Durvalumab; Tremelimumab; Regorafenib; PexaVec; Radiotherapy; Temozolomide; mFOLFOX6; MSI,microsatellite instability; MSI-H,microsatellite instability -high; dMMR,deficient mismatch repair; pMMR,proficient mismatch repair; MSS,microsatellite stable group; MINORS,methodological index for non-randomized studies.

### 3.2 Quality assessment

12 non-randomized studies were assessed using the methodological index for non-randomized studies (MINORS), which categorized studies into three dimensions based on eight items, including stated aim, population election, endpoints, and prospective calculation. The quality assessment details are shown in **Table 2**.

**Table 2:** Methodological index for non-randomized studies (MINORS) for quality.

Studies	A clearly stated aim	Inclusion of consecutive patients	Prospective collection of data	Endpoints appropriate to the aim of the study	Unbiased assessment of the study endpoint	Follow-up period appropriate to the aim of the study	Loss to follow up less than 5%	Prospective calculation of the study size	Scores
André et al., 2022	2	1	2	2	1	2	2	2	14
Chen et al., 2020	2	2	2	2	1	2	2	2	15
Cohen et al., 2020	2	2	2	2	1	2	2	2	15
Fakih et al., 2023	2	2	2	2	1	1	2	2	14
Marie et al., 2021	2	2	2	2	1	2	2	2	15
Monge et al., 2023	2	1	2	2	1	2	1	2	13
Monjazeb et al., 2021	2	1	2	2	1	1	1	2	12
Morano et al., 2022	2	2	2	2	1	1	2	2	14
Parikh et al., 2021	2	1	2	2	1	2	2	2	14
Segal et al., 2021	2	1	2	2	1	1	2	2	13
Thibaudin et al., 2023	2	2	2	2	1	2	2	2	15
Zhao et al., 2023	2	2	2	2	1	2	1	2	14

### 3.3 Efficacy

#### 3.3.1 Survival

**Figure 2:**
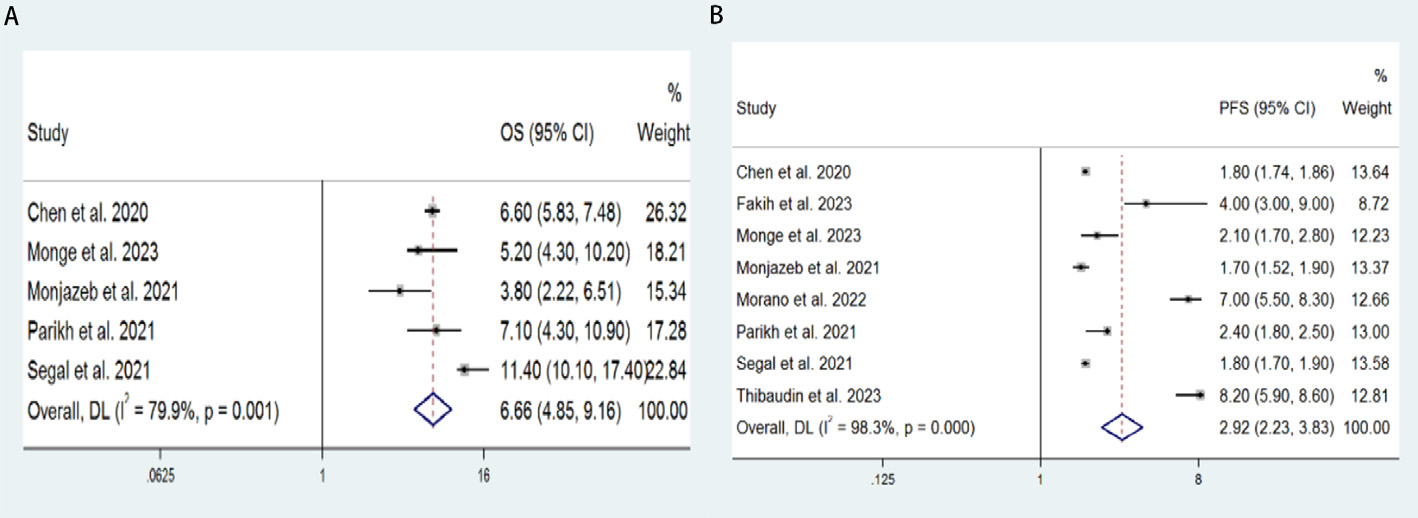
Forest plot for the **(A)** median overall survival (mOS) and **(B)** median progression-free survival (mPFS) in aCRC patients receiving PD-1/PD-L1 inhibitors combined with CTLA-4 inhibitors.

5 studies with a total of 221 patients were included to determine the OS of patients treated with PD-1/PD-L1 inhibitor and CTLA-4 inhibitor. As shown in **Figure 2A**, the random-effect model meta-analysis illuminated that the pooled mOS was 6.66 months (95%CI: 4.85-9.16, I2 = 79.9%, P=0.001), suggesting that PD-1/PD-L1 and CTLA-4 immune checkpoint inhibitors achieved good mOS in the treatment of aCRC. We also analyzed the mPFS of PD-1/PD-L1 inhibitor and CTLA-4 inhibitor in advanced CRC. As shown in **Figure 2B**, the pooled mPFS of 340 patients in 8 studies was 2.92 months (95%CI: 2.23-3.83, I2 = 98.3%, P<0.0001). The result suggests that PD-1/PD-L1 inhibitors and CTLA-4 inhibitors performed well in terms of mPFS in the treatment of aCRC. These results all indicate a PD-1/PD-L1 inhibitors and CTLA-4 inhibitors are to the benefit of aCRC patients’ survival, as supported by the meta-analysis outcomes.

#### 3.3.2 Response rates

**Figure 3:**
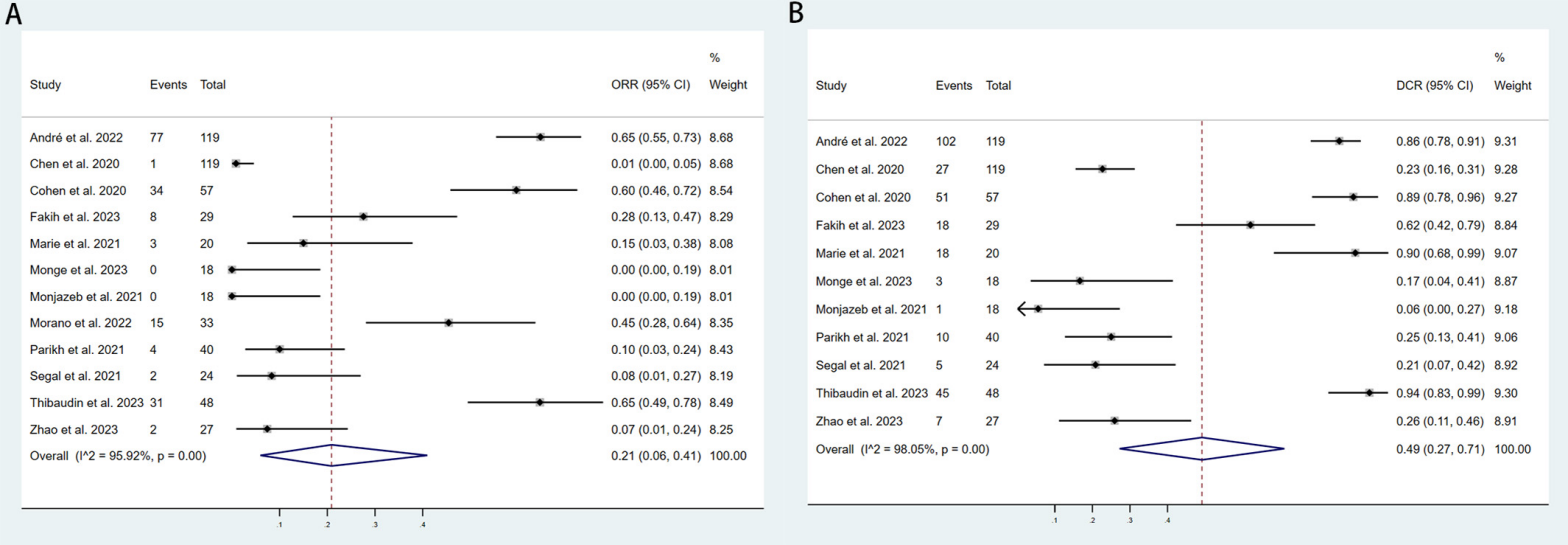
Forest plot for the **(A)** Objective response rate (ORR) and **(B)** Disease control rate (DCR) in aCRC patients receiving PD-1/PD-L1 inhibitors combined with CTLA-4 inhibitors.

We included 12 studies with a total of 566 patients to assess the objective response rate (ORR). The pooled ORR was 21% (95% CI: 6%–41%, I² = 95.92%, P < 0.001, **Figure 3A**). For disease control rate (DCR), we pooled data from 11 studies involving 563 patients, resulting in a DCR of 49% (95% CI: 27%–71%, I² = 98.05%, P < 0.001, **Figure 3B**). Subgroup analyses were conducted based on country (USA vs. Other), sample size (≥40 vs. <40), additional therapy (None vs. Yes), type of immune checkpoint inhibitors (Nivolumab+Ipilimumab, Durvalumab+Tremelimumab, QL1706 [PSB205]), and molecular phenotype (dMMR/MSI-H vs. pMMR/MSS vs. NR) to further explore the efficacy of PD-1/PD-L1 and CTLA-4 inhibitors. As shown in **Table 3**, the ORR for patients treated in the USA was significantly lower compared to patients from other countries. Additionally, studies with sample sizes ≥40 reported better ORR than those with <40 samples. The subgroup of patients who did not receive additional therapies showed higher ORR compared to those who did. Furthermore, the combination of Nivolumab and Ipilimumab demonstrated a higher ORR than both Durvalumab+Tremelimumab and QL1706 (PSB205). The ORR for patients with dMMR/MSI-H was also higher than for those with pMMR/MSS. Similarly, the DCR followed the same pattern. More favorable outcomes were observed in patients from non-USA countries, in studies with sample sizes ≥40, in those not receiving other therapies, in patients treated with Nivolumab+Ipilimumab, and in those with dMMR/MSI-H (**Table 4**). These subgroup differences may be key sources of heterogeneity.

**Table 3:** Subgroup analysis of pooled of the overall response rate.

Subgroup	NO. of studies	Ratio (95% CI)	Heterogeneity	
			I2 (%)	Ph
Country				
USA	7	14% (0-39%)	94.52	<0.001
Other	5	31% (4%-68%)	97.4	<0.001
Sample				
≥40	5	35% (6%-73%)	98.71	<0.001
<40	7	12% (2%-26%)	81.42	<0.001
Other therapy				
None	4	28% (0-73%)	98.46	<0.001
Yes	8	18% (4%-37%)	90.51	<0.001
ICIs				
Nivolumab+Ipilimumab	5	41%(20%-63%)	92.18	<0.001
Durvalumab+tremelimumab	6	10% (0-35%)	95.01	<0.001
QL1706 (PSB205)	1	7% (1%-24%)	–	–
Molecular phenotype				
dMMR/MSI	2	63%(56%-70%)	–	–
pMMR/MSS	8	15%(1%-36%)	94.44	<0.001
NR	2	10%(3%-21%)	–	–

**Table 4:** Subgroup analysis of pooled of the disease control rate.

Subgroup	NO. of studies	Ratio (95% CI)	Heterogeneity	
			I2 (%)	Ph
Country				
USA	7	44%(14%-74%)	97.67	<0.001
Other	4	58%(19%-97%)	98.78	<0.001
Sample				
≥40	5	64% (34%-93%)	98.61	<0.001
<40	6	37% (8%-66%)	95.63	<0.001
Other therapy				
None	4	56% (20%-92%)	98.64	<0.001
Yes	7	45% (13%-77%)	97.91	<0.001
ICIs				
Nivolumab+Ipilimumab	4	41%(20%-63%)	96.09	<0.001
Durvalumab+tremelimumab	6	10% (0-35%)	98.52	<0.001
QL1706 (PSB205)	1	26% (11%-46%)	–	–
Molecular phenotype				
dMMR/MSI	2	87%(82%-92%)	–	–
pMMR/MSS	7	35%(12%-62%)	94.95	<0.001
NR	2	55%(40%-69%)	–	–

### 3.4 Safety

**Figure 4:**
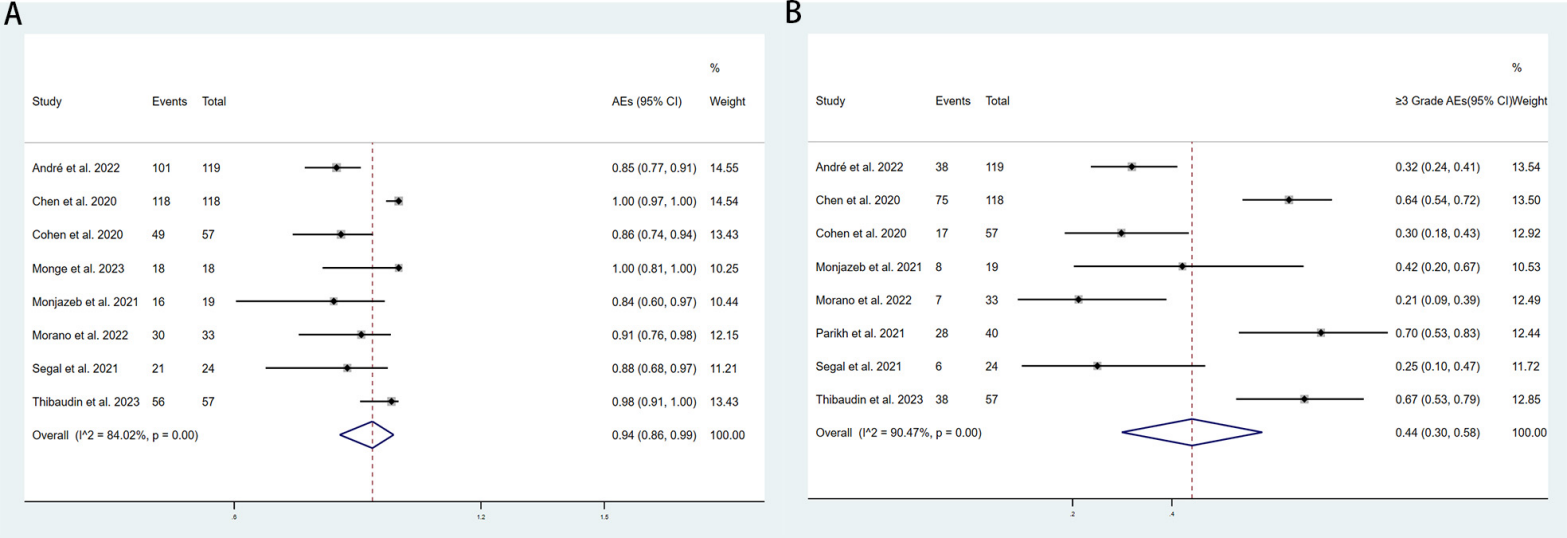
Forest plot for the **(A)** adverse events(AEs) rate and **(B)** and ≥grade 3 AEs rate in aCRC patients receiving PD-1/PD-L1 inhibitors combined with CTLA-4 inhibitors.

The adverse events (AEs) associated with the combination of anti-PD-1/PD-L1 and anti-CTLA-4 in treating aCRC were analyzed, including both all-grade AEs and ≥ grade 3 AEs. Most patients experienced grade 1–2 AEs, which were generally well tolerated. Data from 8 studies reported on the rates of any-grade AEs and ≥ grade 3 AEs. As shown in **Figure 4**, the pooled rate of all-grade AEs was 94% (95% CI: 86%–99%, I² = 84.02%, P < 0.001, **Figure 4A**), while the pooled rate of ≥ grade 3 AEs was 44% (95% CI: 30%–59%, I² = 90.47%, P < 0.001, **Figure 4B**). **Table 5** and **Supplementary Figures S1, S2** indicate that the three most commonly reported AEs were fatigue (26%, 95% CI: 6%–54%), diarrhea (19%, 95% CI: 9%–31%), and anemia (18%, 95% CI: 0%–55%). The most frequent ≥ grade 3 AEs were lymphopenia (5%, 95% CI: 3%–7%), diarrhea (3%, 95% CI: 1%–5%), and increased aspartate aminotransferase (3%, 95% CI: 2%–5%).Subgroup analyses of AEs and ≥ grade 3 AEs were conducted, as shown in **Tables 6** and **7**. The results suggested that country, sample size, additional therapies, immune checkpoint inhibitors (ICIs), and molecular phenotype were potential sources of heterogeneity. Patients in the USA had a lower incidence of both all-grade AEs (90% vs. 96%) and ≥ grade 3 AEs (42% vs. 46%) compared to those from other countries, with lower heterogeneity. Studies with sample sizes ≥ 40 reported higher rates of all-grade AEs (94% vs. 92%) and ≥ grade 3 AEs (52% vs. 27%) than those with sample sizes < 40. Additionally, patients receiving other therapies in combination with PD-1/PD-L1 and CTLA-4 inhibitors had slightly higher rates of both all-grade AEs (94% vs. 93%) and ≥ grade 3 AEs (45% vs. 42%). The type of ICIs used also influenced AE rates: Nivolumab+Ipilimumab had a lower rate of all-grade AEs (86% vs. 97%) compared to Durvalumab+Tremelimumab, with similar trends observed for ≥ grade 3 AEs (38% vs. 51%). Finally, the pMMR/MSS molecular phenotype was associated with a higher incidence of all-grade AEs (95% vs. 85%) and a greater occurrence of ≥ grade 3 AEs (45% vs. 31%).

**Table 5:** Adverse events of the studies included in the meta-analysis.

TRAEs	All Grade	
	ES,%(95%CI)	I^2%^
fatigue	26 (6-54)	96.84
diarrhea	19 (9-31)	86.91
anemia	18 (0-55)	98.37
fever	13 (1-35)	96.17
nausea	12 (0-34)	96.40
pruritus	10 (1-26)	93.59
TRAEs	≥3 Grade	
	ES,%(95%CI)	I^2%^
lymphopenia	5 (3-7)	0
diarrhea	3 (1-5)	7.85
Aspartate aminotransferase increase	3 (2-5)	0
erythra	3 (1-5)	0
anemia	3 (1-5)	0
fatigue	2 (1-4)	0

**Table 6:** Subgroup analysis of pooled of the adverse events.

Subgroup	NO. of studies	Ratio (95% CI)	Heterogeneity	
			I2 (%)	Ph
Country				
USA	4	90% (81%-96%)	46.51	0.13
Other	4	96%(86%-100%)	86.29	<0.001
Sample				
≥40	4	94% (82%-100%)	92	<0.001
<40	4	92% (83%-98%)	32.3	0.22
Other therapy				
None	3	93% (75%-100%)	–	–
Yes	5	94% (87%-99%)	51.23	0.08
ICIs				
Nivolumab+Ipilimumab	3	86% (81%-91%)	–	–
Durvalumab+tremelimumab	5	97% (89%-100%)	76	<0.001
Molecular phenotype				
dMMR/MSI	2	85%(80%-90%)	–	–
pMMR/MSS	5	95%(84%-100%)	80.49	<0.001
NR	1	98%(91%-100%)	–	–

**Table 7:** Subgroup analysis of pooled of the ≥grade 3 adverse events.

Subgroup	NO. of studies	Ratio (95% CI)	Heterogeneity	
			I2 (%)	Ph
Country				
USA	4	42% (22%-63%)	87.29	<0.001
Other	4	46% (24%-68%)	93.17	<0.001
Sample				
≥40	5	52% (35%-70%)	92.46	<0.001
<40	3	27% (16%-38%)	–	–
Other therapy				
None	3	42% (20%-64%)	–	–
Yes	5	45% (24%-67%)	89.84	<0.001
ICIs				
Nivolumab+Ipilimumab	4	38% (20%-56%)	89.39	<0.001
Durvalumab+tremelimumab	4	51% (33%-69%)	84.53	<0.001
Molecular phenotype				
dMMR/MSI	2	31%(25%-38%)	–	–
pMMR/MSS	5	45%(25%-65%)	87.85	<0.001
NR	1	67%(53%-79%)	–	–

## 4 Discussion

Chemotherapy, cytotoxic drugs, and molecular targeted therapies have commonly been used in the treatment of advanced colorectal cancer (aCRC), but their efficacy has plateaued (14, 53). Recently, numerous clinical trials related to immunotherapy have confirmed that it is an encouraging new treatment strategy for colorectal cancer (54–57). The two most widely studied immune checkpoints are PD-1 and CTLA-4. PD-1 is a cell surface receptor commonly found on T cells, B cells, and NK cells. When the PD-1 receptor binds to its ligand, it inhibits cell proliferation, cytokine secretion, and the cytotoxic capabilities of immune cells, thus weakening the immune response (58). CTLA-4 receptors, expressed on activated or regulatory T cells, bind to B7 ligands on antigen-presenting cells with a higher affinity and lower surface density. This binding prevents CD28 receptors from interacting with B7 ligands, leading to a net downregulation of T cell activation (59). A previous meta-analysis indicated that PD-1 inhibitors and combination immunotherapy demonstrate promising clinical responses and overall survival (OS) rates, alongside manageable adverse events (AEs) in the treatment of dMMR colorectal cancer (60). Although single-agent immune checkpoint inhibitors (ICIs) perform well, one study noted that ICI therapy resistance has been observed in up to 50% of patients with microsatellite instability-high (MSI-H) CRC. Furthermore, single-agent ICIs show minimal efficacy in patients with proficient mismatch repair (pMMR) or microsatellite stable (MSS) metastatic CRC (61, 62). To address tumor escape mechanisms, substantial efforts have been made to enhance the clinical efficacy of ICIs in colorectal cancer through combination therapies. Specifically, the combination of CTLA-4 and PD-1/PD-L1 blockade increases T cell activation and treatment efficacy, resulting in better antitumor outcomes (56, 63). Preclinical studies have shown that combined immunotherapy is superior to monotherapy. The dual blockade of PD-1/PD-L1 and CTLA-4 can increase the number of T cells regulated by multiple mechanisms and enhance their effector function (64). Currently, extensive research is being conducted to extend the indications for dual immune checkpoint blockade with PD-1 and CTLA-4 to various solid tumors (64, 65). However, the efficacy and safety of PD-1/PD-L1 and CTLA-4 immune checkpoint inhibitors in the treatment of aCRC remain largely unconfirmed. Twelve clinical trials involving 566 patients with metastatic colorectal cancer (mCRC) have reported results for these agents. Therefore, we conducted a meta-analysis to provide valid and reliable conclusions. We compared the effectiveness and AEs of several therapy regimens involving PD-1/PD-L1 and CTLA-4 immune checkpoint inhibitors for potential clinical application. The pooled objective response rate (ORR), disease control rate (DCR), and overall AE rate were 50%, 33%, 94%, and 44%, respectively. The pooled median overall survival (mOS) and median progression-free survival (mPFS) were 8.81 months and 2.57 months.

In order to further analyze the efficacy and safety of PD-1/PD-L1 and CTLA-4 immune checkpoint inhibitors, we performed subgroup analyses based on country (USA, Other), sample size (≥40, <40), other therapies (None, Yes), types of ICIs (Nivolumab+Ipilimumab, Durvalumab+tremelimumab, QL1706 (PSB205)), and molecular phenotypes (dMMR/MSI-H, pMMR/MSS, NR). In terms of country, the USA exhibited lower ORR and DCR, as well as fewer AEs and grade ≥3 AEs compared to other countries. Regarding sample size, when the sample size is ≥40, this may indicate higher ORR, DCR, and an increased incidence of AEs and grade ≥3 AEs. Concerning ICIs types, Nivolumab+Ipilimumab is considered to have higher efficacy and safety than Durvalumab+tremelimumab and QL1706 (PSB205). From the molecular phenotype perspective, the efficacy of dual ICIs in pMMR/MSS aCRC patients is lower than that in dMMR/MSI-H aCRC patients, while the incidence of adverse events may be higher. This discrepancy arises because MSI-H tumors typically have a high tumor mutation burden (TMB) and a high tumor neoantigen burden (TNB), which can activate the immune system more effectively. In contrast, MSS tumors have a lower mutation burden and generate relatively fewer neoantigens, reducing the likelihood of T cells recognizing and attacking tumor cells (4, 61). Notably, the combination of different treatment modalities is often believed to enhance therapeutic effects. However, our analysis yielded opposite results. When PD-1/PD-L1 and CTLA-4 immune checkpoint inhibitors are combined with other treatments (such as chemotherapy, radiotherapy, etc.), the ORR and DCR are significantly lower, and the risk of adverse effects is higher. This may be due to the limited number of studies that we did not categorize by different treatment regimens. However, a careful review of the literature reveals that the efficacy of PD-1/PD-L1 and CTLA-4 immune checkpoint inhibitors combined with chemotherapy is better than the pooled results. Past studies have demonstrated that cytotoxic chemotherapy could reduce immunosuppressive T-regulatory cells, induce cell surface death receptor density to promote immune killing, and enhance the release of neoantigens in the microenvironment (66–68). In the Thibaudin study, a median PFS of 8.2 months and an objective response rate of 63% were reported for the treatment plan involving durvalumab and tremelimumab combined with mFOLFOX6, which compares favorably with FOLFOX monotherapy (44). This study supports the notion that chemo-immunotherapy could promote immune responses against shared tumor antigens and neoantigens in MSS metastatic CRC, with this immune response associated with therapeutic efficacy. In another study, temozolomide in combination with ipilimumab and nivolumab in patients with microsatellite stabilization achieved encouraging results, with median PFS and mOS of 7.0 and 18.4 months, respectively, and an overall response rate of 45% (47). This provides evidence of the role of temozolomide as an immune-sensitizing agent for MSS and immune-cold aCRCs. However, the combination of radiotherapy with CTLA-4 and PD-1 blockers for the treatment of aCRC was less than satisfactory. Although preclinical data suggest that radiation can induce tumor-specific immune responses (69), increase T-cell infiltration (70), and promote immune-mediated tumor cell death (71), clinical trials to date have not met their intended goals. In a study involving 40 aCRC patients receiving nivolumab and ipilimumab in combination with radiation therapy, Parikh observed a DCR of 25% and an ORR of 10% (48), significantly lower than those reported in previous studies that did not involve radiation therapy. Two other similar clinical trials combining PD-L1 and CTLA-4 inhibitors with radiation therapy in aCRC patients also failed to meet their predefined endpoints, although they noted that this treatment can enhance the immunogenicity of the local and systemic immune microenvironment (36, 49). In the future, more clinical trials are warranted to investigate the timing factors associated with radiation in immunotherapy and to identify appropriate radiation dosages (72, 73).

The results of this meta-analysis demonstrate that the combination of PD-1 inhibitors and CTLA-4 inhibitors has shown promising and profound clinical responses in clinical studies. The synergistic effect of combining PD-1/PD-L1 and CTLA-4 inhibitors surpasses the sum of their individual contributions as monotherapies (74, 75). Thibaudin reported that the median progression-free survival (mPFS) for metastatic colorectal cancer treated with durvalumab and tremelimumab in combination with FOLFOX was 8.2 months, significantly higher than the mPFS of 5-6 months for FOLFOX alone and comparable to the mPFS of 8 months for the bevacizumab-combined chemotherapy regimen (44, 76). A previous meta-analysis of PD-1/PD-L1 inhibitors in the treatment of aCRC suggested that the objective response rate (ORR) for dMMR/MSI-H aCRC was 37%, while that for pMMR/MSS aCRC was 11% (77). In our molecular phenotype subgroup analysis, the ORR for PD-1/PD-L1 inhibitors combined with CTLA-4 inhibitors in dMMR/MSI-H aCRC was 63%, whereas in pMMR/MSS aCRC it was 18%, both significantly higher than the 37% and 11% observed for PD-1/PD-L1 inhibitors alone, respectively. Treatment-related adverse events (TRAEs) are associated with overactivation of the immune system, leading to damage to one or more organs. As expected, we observed that the incidence of TRAEs in patients receiving the combination of anti-PD-1/PD-L1 and anti-CTLA-4 therapy was higher compared to those receiving anti-PD-1/PD-L1 monotherapy (94% vs. 85%) (36, 77). Among the included studies, the highest incidence of grade ≥3 TRAEs was reported by Parikh (48). Notably, despite a high incidence of grade ≥3 TRAEs in this study, only a few patients discontinued treatment due to clinically relevant toxicity. Similar results were observed in other studies. Moreover, we found that grade ≥3 TRAEs often occurred in the early stages of treatment, and most TRAEs could be controlled and resolved with a short interruption of treatment and a brief course of oral steroid therapy (61, 78). Currently, the incidence of TRAEs is common in anti-PD-1/PD-L1 + anti-CTLA-4 combination therapy, although the pathogenesis is poorly understood (79). We believe that the occurrence of grade ≥3 TRAEs may be associated with drug dosage, treatment cycles, and the patients’ physical conditions. In anti-PD-1/PD-L1 + anti-CTLA-4 combination therapy, it is crucial to enhance monitoring of immunotherapy during treatment, pay attention to severe TRAEs, and manage them according to relevant guidelines.

## 5 Limitations

There are several limitations in the current meta-analysis. First, most studies had small sample sizes. Additionally, there is currently limited data on the combination therapy of PD-1 inhibitors and CTLA-4 inhibitors, and few studies are randomized or blinded. Second, we relied on published trial data but did not have access to raw patient data, which may introduce biases in our analysis. Therefore, more large-scale clinical trials are needed to further validate the efficacy and safety of the combination therapy of PD-1 inhibitors and CTLA-4 inhibitors. Third, it is crucial to identify the predominant factors influencing immunosuppressive therapy. Due to the limited experimental data, this study did not analyze the associations between gender, age, tumor mutation burden (TMB), tumor neoantigen burden (TNB), and the site of metastasis with treatment efficacy and safety.

## 6 Conclusion

In summary, our meta-analysis demonstrates the efficacy and safety of combination therapy with PD-1 inhibitors and CTLA-4 inhibitors in patients with aCRC, supporting its potential for future clinical application. However, due to the limited clinical data available, further large-scale, multicenter randomized controlled trials (RCTs) are necessary to confirm these findings.

## Data Availability

The original contributions presented in the study are included in the article/**Supplementary Material**. Further inquiries can be directed to the corresponding author.
