# Supplementary material for: Efficacy and safety of PD-1/PD-L1 and CTLA-4 immune checkpoint inhibitors in the treatment of advanced colorectal cancer: a systematic review and meta-analysis
Source: Front Immunol. 2024 Nov 1;15:1485303. doi: 10.3389/fimmu.2024.1485303 (PMC11563947; doi:10.3389/fimmu.2024.1485303)
Supplement: Supplementary file 1 [file DataSheet1.docx]

1. (B) (C)


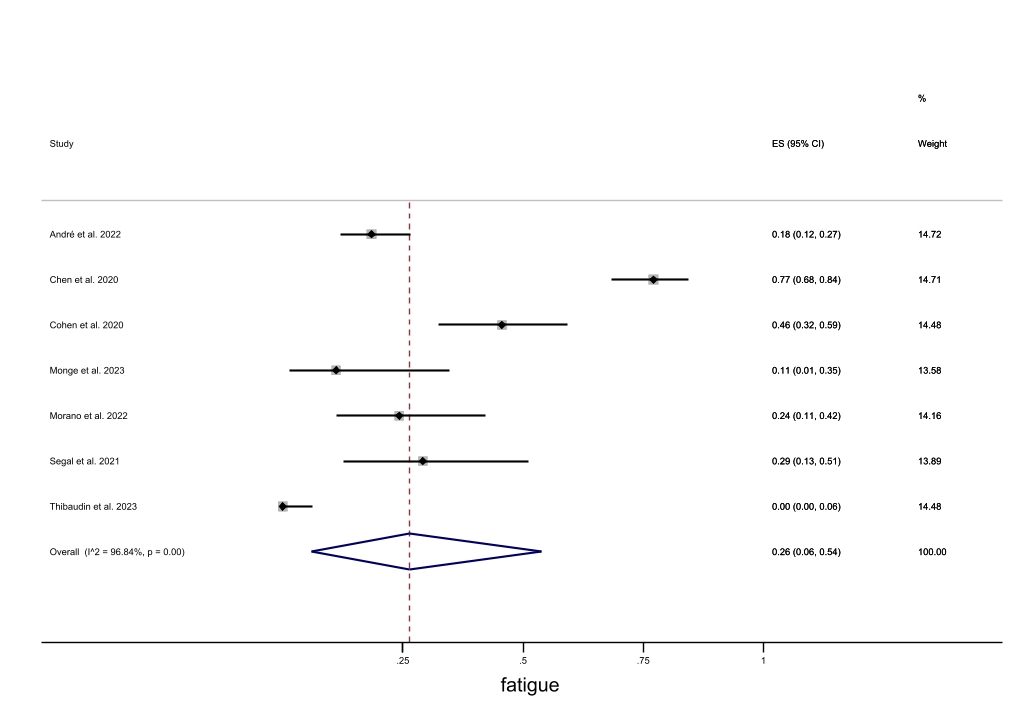

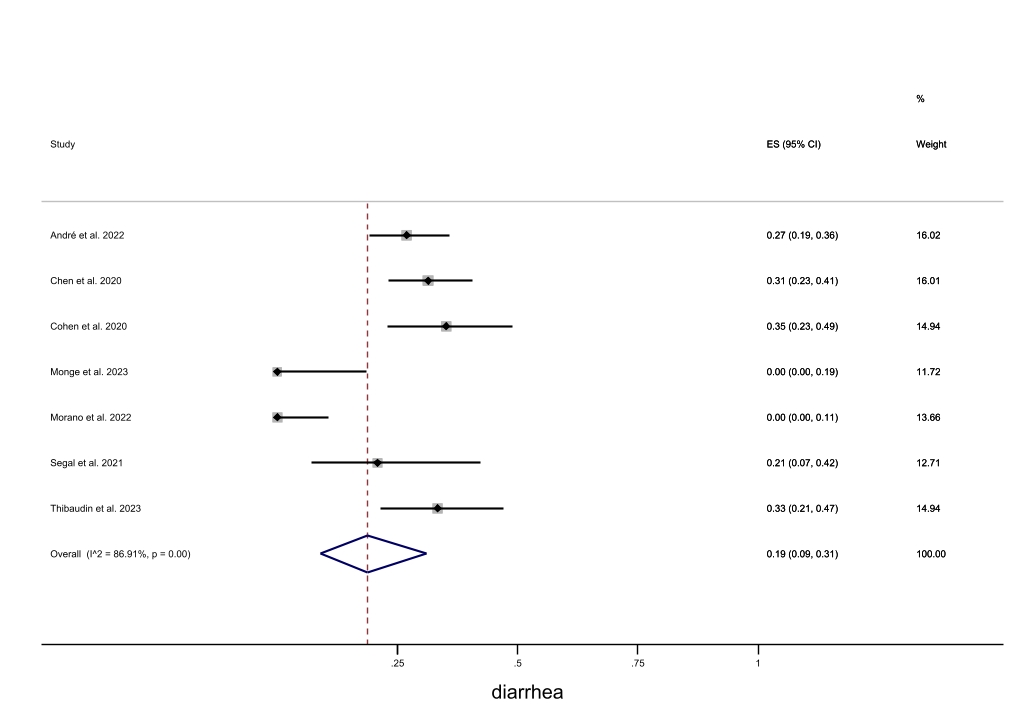

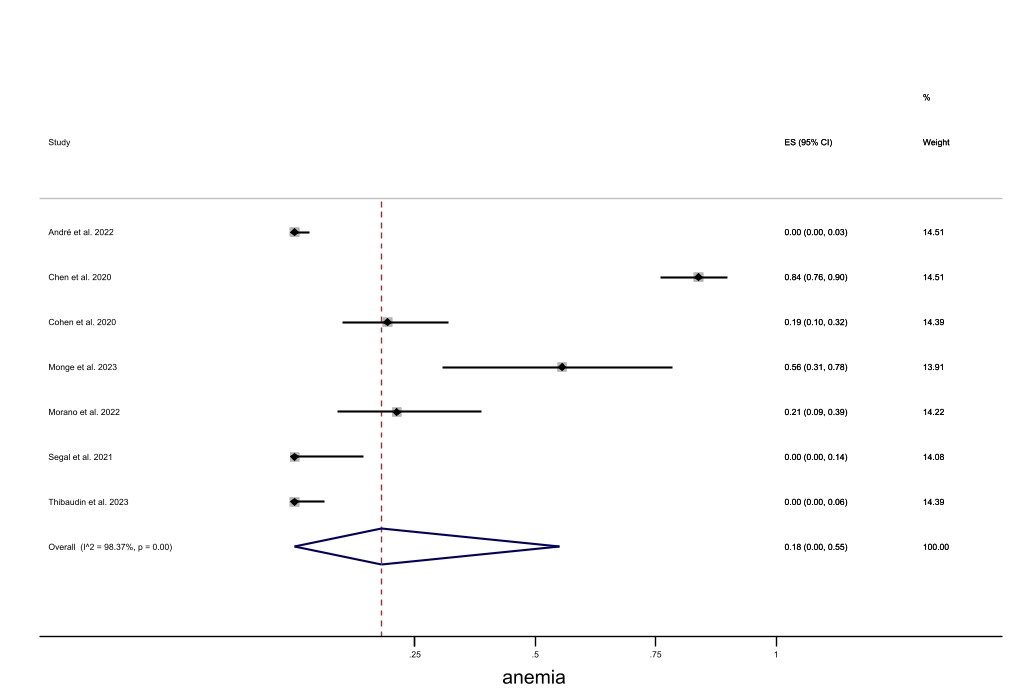


1. (E) (F)


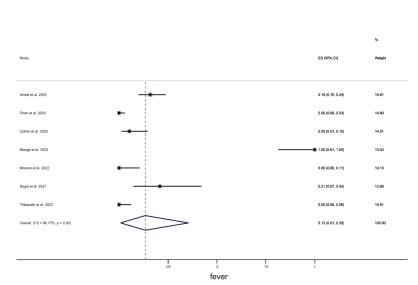

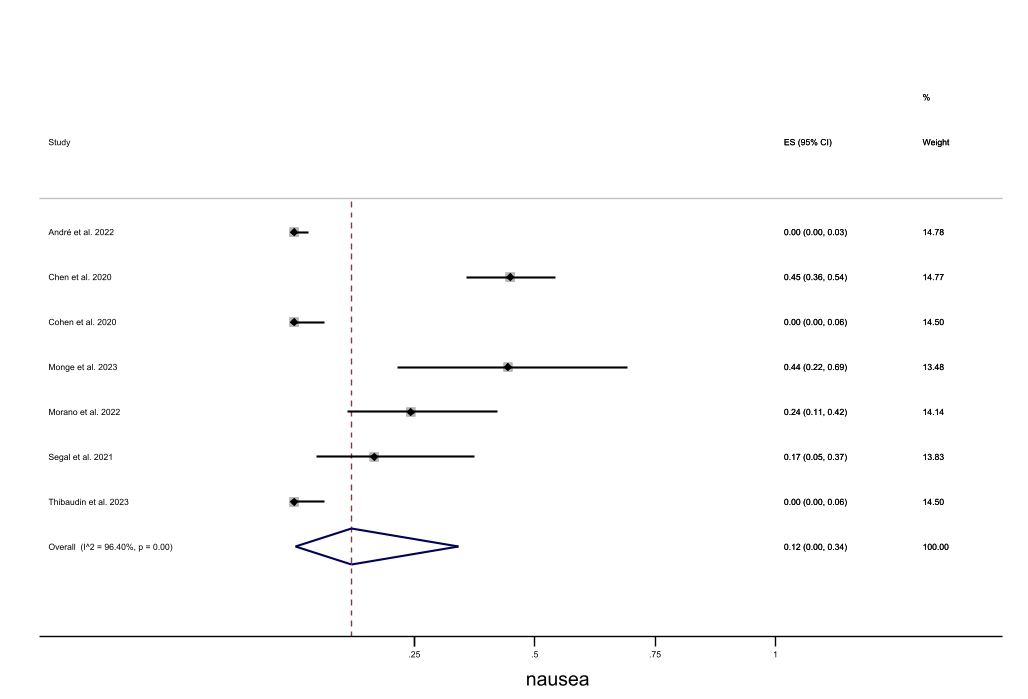

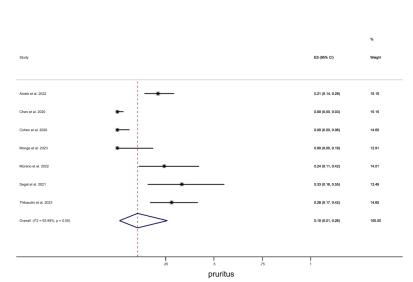


Figure. S1 The forest plot about the pooled results of All Grades (A) fatigue (B) diarrhea (C) anemia (D) fever (E) nausea (F) pruritus

1. (B) (C)


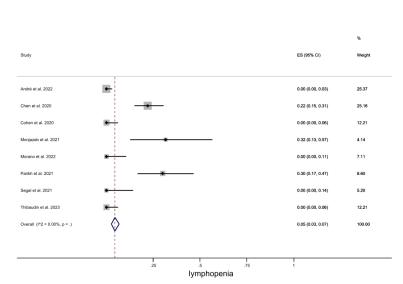

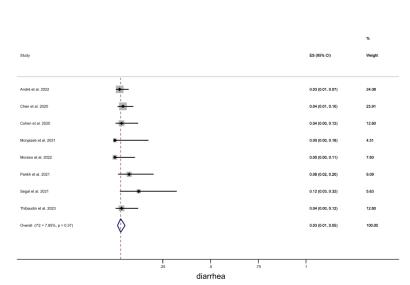

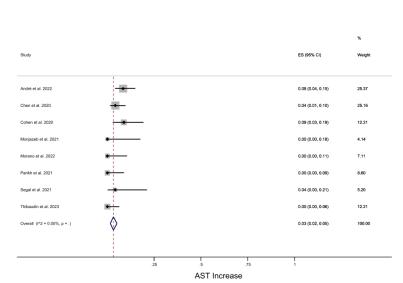


(D) (E) (F)


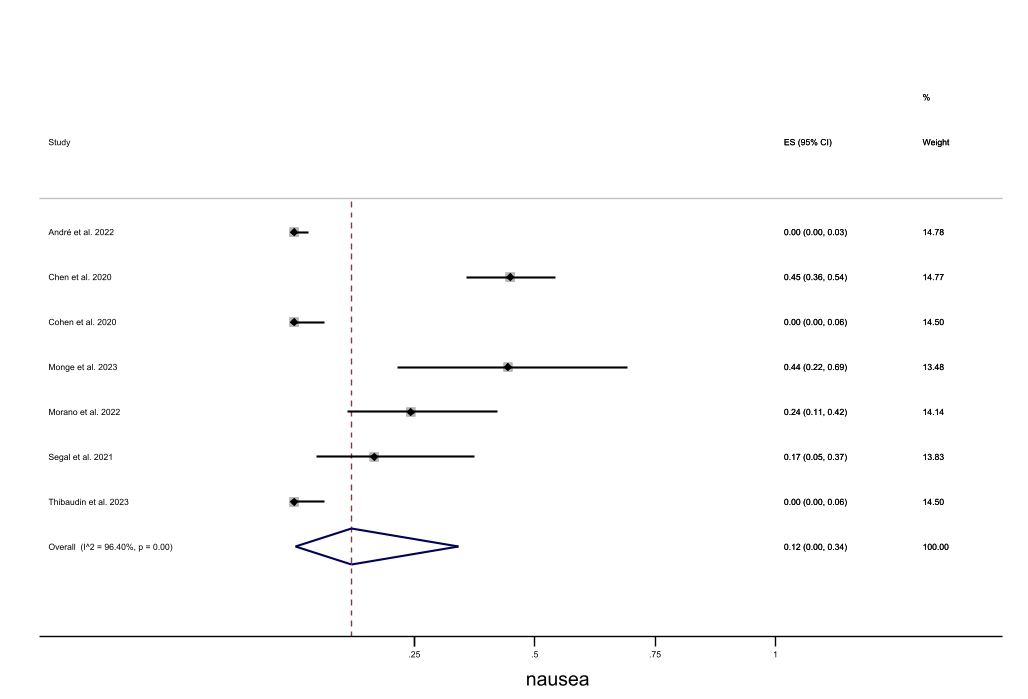

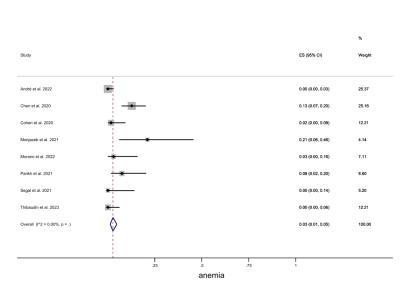

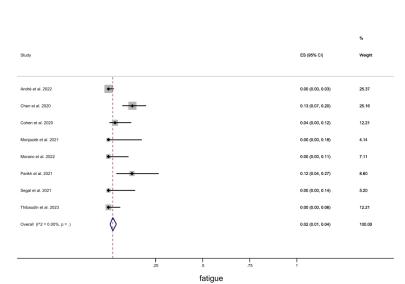


Figure. S2 The forest plot about the pooled results of Grade III and higher adverse events. (A) lymphopenia (B) diarrhea (C) Aspartate aminotransferase increase (D) erythra (E) anemia and (F) fatigue

1. (B) (C)


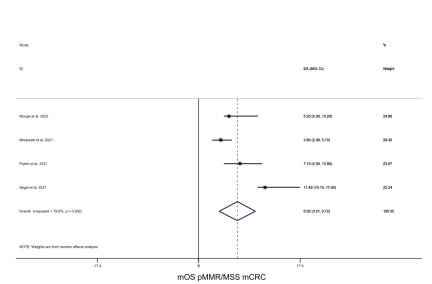

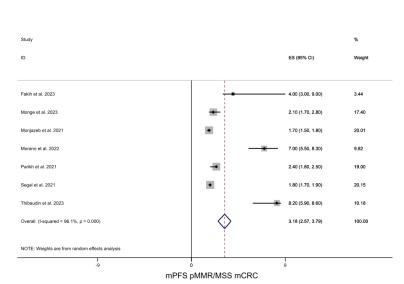

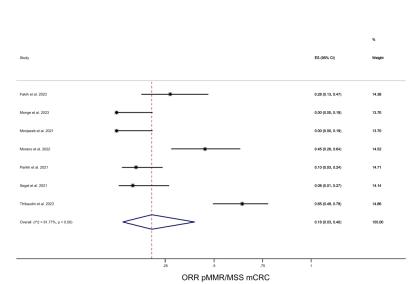


1. (E) (F)


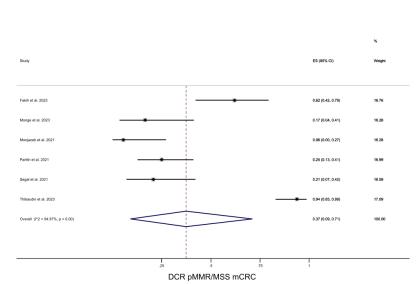

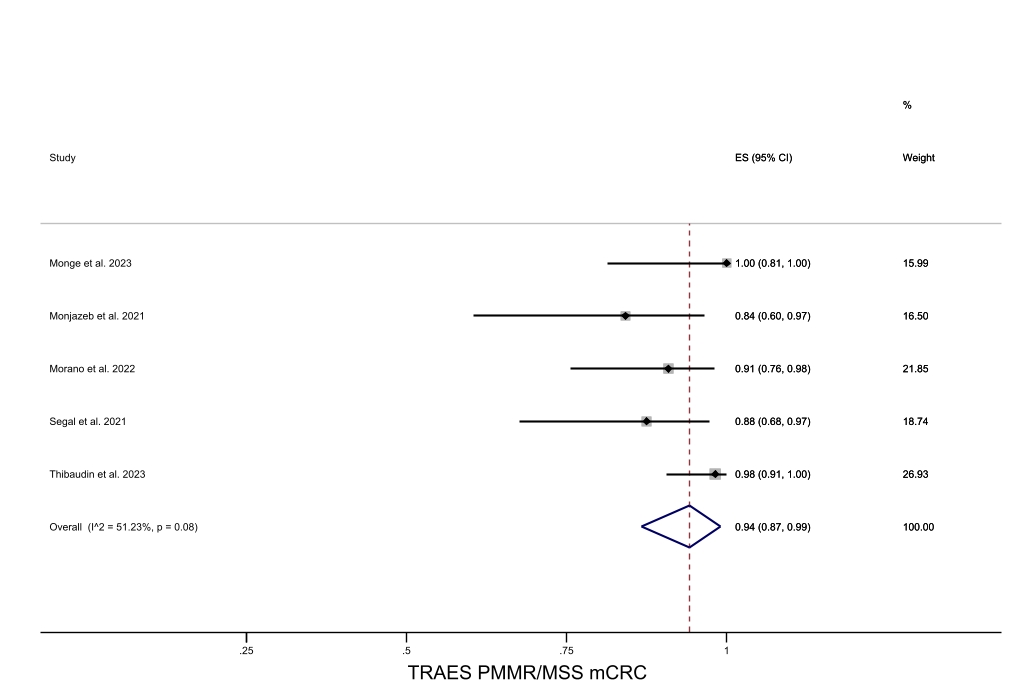

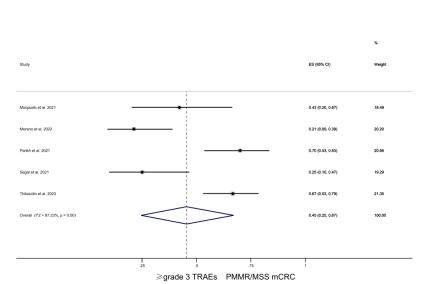


Figure. S3 The forest plot about the pooled results of pMMR/MSS mCRC. (A) mOS (B) mPFS (C) ORR (D) DCR (E) TRAEs and(F) ≥grade 3 TRAEs
